# Unimodal and Multimodal Deep Learning for Pressure Injury Identification: Scoping Review

**DOI:** 10.2196/91048

**Published:** 2026-08-10

**Authors:** Yingxue Sun, Congcong Liu, Ji-cheng Zhang, Zhengang Wei, Zhenfeng Zhou, Chunmei Fan, Fangyan Yue

**Affiliations:** 1 Department of Critical Care Medicine (Ward 2), Shandong Provincial Hospital Affiliated to Shandong First Medical University, No. 324, Jingwu-Weiqu Road, Huaiyin District, Jinan, Shandong Province, 250021, China, 86 15863545853

**Keywords:** deep learning, pressure injuries, intelligent recognition, staging, assessment, artificial intelligence, AI

## Abstract

**Background:**

Pressure injury (PI) can cause severe infections or death, and has a high global prevalence. Accurate staging is vital for effective intervention. Deep learning streamlines PI assessment; enhances efficiency; and yields practical, accurate results.

**Objective:**

The primary objective of this scoping review was to map the landscape of deep learning applications for PI identification and categorize the existing body of evidence into distinct technological trajectories: unimodal imaging and multimodal integration.

**Methods:**

We searched the following databases and sources: PubMed, the Cochrane Library, IEEE Xplore, and Web of Science. This scoping review was conducted in accordance with the PRISMA-ScR (Preferred Reporting Items for Systematic Reviews and Meta-Analyses extension for Scoping Reviews) guidelines.

**Results:**

A total of 15 articles were included: 40% (n=6) studies applied multimodal integration, whereas 60% (n=9) applied unimodal imaging. In total, 26 models were involved. Different models exhibited varying accuracy rates in staging PI, with overall accuracy fluctuating between 54.8% and 93.7%. The same model demonstrated significant variations in recognition accuracy across different studies. Multimodal integration appeared to be a distinguishing characteristic of the studies that will progress toward clinical validation.

**Conclusions:**

The current landscape of deep learning in PI identification is heavily skewed toward unimodal imaging, creating a modality gap that hinders clinical translation. While unimodal models demonstrate high accuracy in controlled settings, future research must prioritize bridging the gap from unimodal imaging to true multimodal integration to achieve clinical-grade reliability.

## Introduction

Pressure injury (PI) refers to localized damage to the skin and/or underlying tissues resulting from intense and/or prolonged pressure or pressure combined with shear forces [1]. The affected areas typically occur over bony prominences or at points of contact between the skin and medical devices. The prevalence of PI has consistently remained at a high level across different countries and regions [2]. A meta-analysis revealed that the prevalence of PI among hospitalized adults worldwide between 2008 and 2018 was 12.8% [3]. Advanced-stage PI typically proves difficult to heal within a short time frame. This prolongs hospital stays by 3 to 5 times and increases mortality risk by 2 to 3 times [4]. PI may lead to severe infections or even death, posing significant challenges to both patients and health care systems.

Accurate diagnosis of PI staging is a prerequisite for medical intervention, and nurses’ assessment of PI staging directly influences treatment outcomes. In PI nursing, junior nurses may misdiagnose staging accuracy due to insufficient expertise, assessment skills, and systematic training, compounded by constraints in resources and psychological support [5]. Research indicates that the accuracy of PI staging diagnosis ranges from merely 30% to 70%. This may be attributable to several factors: PI staging relies on evaluators’ subjective descriptions of wound tissue appearance, with considerable variation in diagnostic interpretation between assessors; achieving accurate staging requires extensive accumulated experience; and the unclear demarcation between the injured area’s coloration and normal skin may also contribute [6]. Therefore, advanced technology is required to assist medical personnel in PI staging diagnosis, thereby enhancing the accuracy and efficiency of PI classification.

The vast majority of existing deep learning research has focused on unimodal imaging. While architectural evolutions, from convolutional neural networks (CNNs) to vision transformers, have achieved high accuracy in laboratory settings, these models remain fundamentally constrained by the limitations of visible light [7]. To overcome these limitations, the field is gradually shifting toward multimodal integration. A research problem or machine learning model is described as multimodal when it needs to incorporate and process at least 2 distinct modalities (such as visual data, audio data, tactile data, and text) simultaneously. Multimodal fusion inherently encompasses not only data-level integration but also feature-level fusion, where heterogeneous representations derived from the same or different sources are combined to provide complementary information [8]. Current research is beginning to explore the multimodal fusion of wound images and clinical signs to assess the depth of damage to the underlying tissue [9]. The existing literature remains insufficient in terms of verifying the generalization ability, adaptability to small sample sizes, and multicenter consistency of multimodal models in real-world clinical settings; model performance is prone to significant fluctuations [10]. Several reviews have examined the use of intelligent recognition models for wound assessment, including the comprehensive diagnosis of diabetic foot ulcers, arterial ulcers, venous leg ulcers, PIs, and surgical wounds [11]. Some studies have also focused on the use of AI in monomodal image analysis for wound assessment [12]. This raises several questions: what is the current distribution of deep learning studies on PI identification? Specifically, how many studies fall into the category of unimodal vs true multimodal approaches? What are the models for intelligent PI identification, what are the training methods, and how effective are they? The primary objective of this scoping review was to map the landscape of deep learning applications for PI identification, categorize the existing body of evidence into distinct technological trajectories (unimodal imaging and multimodal integration), evaluate the translational readiness of these studies, and assess their validation strategies and clinical implementation stages.

## Methods

### Protocol and Registration

This review was based on the recommendations of the PRISMA-ScR (Preferred Reporting Items for Systematic Reviews and Meta-Analyses extension for Scoping Reviews) guidelines [13] and used the methodological framework developed by Peters et al [14]. The completed PRISMA-ScR checklist is provided in Checklist 1. This study was registered with PROSPERO on December 12, 2025 (registration number CRD420251251573).

### Eligibility Criteria

The inclusion criteria were defined using the population, concept, and context framework. Detailed inclusion and exclusion criteria can be found in Multimedia Appendix 1.

### Information Sources

To identify potentially relevant studies, the following bibliographic databases were searched up to December 10, 2025: PubMed, Cochrane Library, IEEE Xplore, and Web of Science. We limited the search to English-language publications and included specific article types (journal articles and conference proceedings), explicitly excluding preprints. The search strategies were drafted by an experienced librarian and further refined through team discussion. We supplemented our search with forward and backward citation chasing to minimize the risk of missing key studies.

### Search

This review was conducted by combining the terms “deep learning,” “multi-modal,” “artificial intelligence,” “convolutional neural networks,” and “pressure injury.” The search was conducted on April 15, 2026 (see Multimedia Appendix 2 for the complete database search strategies).

### Selection of Sources of Evidence

Two researchers conducted the literature search using the above-mentioned search terms. The final search results were exported into EndNote (Clarivate Analytics), and duplicates were removed by a researcher. Two independent investigators screened the retrieved studies based on their titles and abstracts against the eligibility criteria. Subsequently, the same 2 researchers conducted full-text screening.

### Data Charting Process

We extracted data using the framework proposed by Peters et al [14] and populated data extraction forms. The 2 reviewers independently charted the data, resolving discrepancies through discussion, and continuously updated the data charting form in an iterative process.

### Data Items

The following data were extracted from each included study: article identifiers (author, year, and country), dataset size and source, modality type, model type, task, validation type, and primary metrics.

### Critical Appraisal of Individual Sources of Evidence

According to the methodological framework outlined in the PRISMA-ScR guidelines, this study did not undertake a formal critical appraisal or risk-of-bias assessment of individual studies. The primary aim of a scoping review is to map the breadth and nature of the existing evidence rather than synthesize effect sizes or assess the effectiveness of interventions. Therefore, the inclusion of studies of varying methodological quality, including those of lower quality, was intended to contribute to a comprehensive overview of the current state of research in this field.

### Synthesis of Results

We synthesized the studies according to their dataset size and source, modality type, model type, task, validation type, and primary metrics.

### Ethical Considerations

This scoping review used only previously published secondary data and did not involve direct human participant interaction.

## Results

### Selection of Sources of Evidence

**Figure 1.**
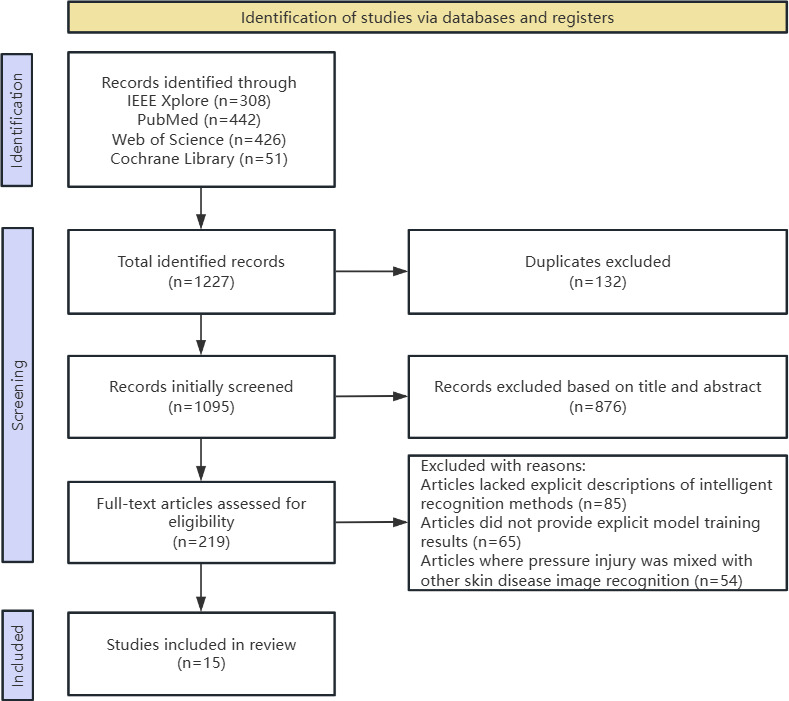
PRISMA (Preferred Reporting Items for Systematic Reviews and Meta-Analyses) flow diagram.

The scoping review process is detailed in the flow diagram shown in Figure 1, presenting the stepwise filtering and the number of studies included at each step.

### Characteristics of the Sources of Evidence

Table 1 presents an overview of the 15 included studies. Regarding study design, all studies were retrospective. Geographically, 33.3% (n=5) of the studies were conducted in China; 20% (n=3) were from South Korea; 13.3% (n=2) were from Turkey; and 6.7% (n=1) each were from the United Kingdom, the United States, Germany, Japan, and Saudi Arabia.

**Table 1.** Characteristics of the included studies (N=15).

Study	Country	Dataset size and source	Modality type	Model	Task	Validation type	Primary metrics^a^
Lei et al [15], 2025	China	7677 images; single center	Unimodal images	AlexNet, VGGNet16, ResNet18, and DenseNet121	Classification	Internal	The classification performance of AlexNet, VGGNet16, ResNet18, and DenseNet121 yielded overall *F*_1_-scores of 94.7%, 88.2%, 98.2%, and 97% (NR^b^) and overall accuracies of 87.7%, 82.4%, 92.4%, and 93.7% (NR), respectively.
Huang et al [16], 2025	China	883 images; CongresoEnfermeria dataset; single center; public	Unimodal images	SE-Swin Transformer and Cascade R-CNN^c^	Classification+detection	External	The Cascade R-CNN architecture achieved an mAP^d^ of 80.9% (NR). The model achieved an accuracy of 87.1% (NR), surpassing other feature extraction models by at least 0.5 percentage points. Results indicate that Cascade R-CNN, with an accuracy of 86.6% (NR), outperformed Faster R-CNN, which achieved 82.5% (NR) accuracy.
Cho and Yoo [17], 2025	South Korea	16,850 images (HAM10000 dataset); multicenter; public data	Unimodal images	ViT^e^	Classification	External	The results showed accuracy and *F*_1_-score values of 87.9% and 77.2% for ViT, 82.8% and 76.3% for ViTMixup, 93.9% and 76.4% for sViT, and 95.6% and 77% for PUC-ViT, respectively (NR).
Brehmer et al [18], 2025	Germany	763 images; multicenter	Multimodal images+patient data	ConvNextV2, EfficientNetV2, and TinyViT	Classification	Internal	The transformer-based TinyViT model demonstrated optimal performance in binary classification for PU^f^, achieving an *F*_1_-score of 93.2% (NR).
Kimura et al [19], 2025	Japan	1007 images; single center	Unimodal images	CCM^g^ and BCM^h^	Classification	Internal	The overall metrics for CCM were accuracy of 81.4% and *F*_1_-score of 80.8% (NR). BCM achieved an accuracy of 87.1% and *F*_1_-score of 85% (NR).
Wang et al [20], 2024	China	1519 images; multicenter	Multimodal images+text	ResNext+wFPN	Classification	Internal	The accuracy of the phase 3 PI^i^ increased from 60.3% to 76.2%. The accuracy of stage 1, 2, and 4 PI were 87%, 78.8%, and 84.5% (NR), respectively. The network’s overall accuracy, precision, recall, and *F*_1_-score were 81.5%, 80.8%, 81.6%, and 81.1% (NR), respectively.
Gui et al [21], 2024	China	1385 images; single center	Multimodal images+heat map	SE-Inception	Classification	Internal	The SE-Inception model demonstrated exceptional accuracy (93%) and AUC^j^ (94%; NR).
Zalluhoğlu et al [22], 2024	Turkey	1202 images; single center	Multimodal images+3D architecture data	Fused CNN	Classification	Internal	Experimental results with a test accuracy of 93%, precision of 93%, recall of 92%, and *F*_1_-score of 93% (NR).
Swerdlow et al [23], 2023	United States	969 images; multicenter	Unimodal images	Mask-R-CNN	Classification+segmentation	Internal	The overall classification accuracy of the Mask-R-CNN model was 92.6% (NR), with a segmentation accuracy of 93% (NR). The *F*_1_-scores for stages 1-4 were 84.2%, 94.7%, 90.7%, and 94.4% (NR), respectively. The Dice coefficients for stages 1-4 were 92%, 85%, 93%, and 91% (NR), respectively.
Kim et al [24], 2023	South Korea	3098 images; multicenter	Unimodal images	SE-ResNext101	Classification	External	The model achieved top-1 accuracy scores of 79.3% and 71.7% (NR) and *F*_1_-scores of 79.1% and 71.5% (NR) on the internal and external test sets, respectively.
Fergus et al [25], 2023	United Kingdom	4290 images; single center	Unimodal images	Faster R-CNN	Classification	Internal	The research findings used a confidence score threshold of 75% (NR), yielding an average precision of 67.9% (NR), a recall of 69.9% (NR), and an *F*_1_-score of 67.8% (NR).
Seo et al [26], 2023	South Korea	2464 images; single center	Unimodal images	VGGNet16, ResNet50, ResNet152, DenseNet201, and EfficientNet-B4	Classification	Internal	For EfficientNet-B4, the macro–*F*_1_-score was calculated to be 89.4% (NR), and the average performance of 2 experienced nurses was reported as 87.8% (NR). EfficientNet-B4 achieved an accuracy of 91.4% (NR). The classification performance of ResNet152, VGGNet16, ResNet50, and DenseNet201 yielded overall accuracies of 84.1%, 83.7%, 82.9%, and 82.1% (NR), respectively. EfficientNet demonstrated superior accuracy to that of nurse B (86.3%; NR) in classifying PIs, although it remained inferior to that of nurse A (94.2%; NR), who had 19 y of experience.
Aldughayfiq et al [27], 2023	Saudi Arabia	Medetec image database (≥1000 images)	Unimodal images	YOLOv5	Detection	External	The YOLOv5 model achieved an overall mAP of 76.9% (NR) on the validation set.
Liu et al [28], 2022	China	528 images; single center	Multimodal images+clinical information	Inception-ResNet-v2	Classification	Internal	In the classification task of erythematous vs nonerythematous wounds, the Inception-ResNet-v2 model achieved an accuracy of 98.5% (NR) and an *F*_1_-score of 94.1% (NR). In the classification task of necrotic tissue, the model attained an accuracy of 97% (NR) and an *F*_1_-score of 93.5% (NR).
Ay et al [29], 2022	Turkey	1091 images from the Pressure Injury Image Dataset; public	Multimodal images+clinical information	DenseNet121, InceptionV3, MobilNetV2, ResNet50, ResNet152, and VGGNet16	Classification	Internal	DenseNet121 achieved a mean AUC of 91% (NR), InceptionV3 achieved a mean AUC of 85% (NR), MobilNetV2 achieved a mean AUC of 85% (NR), ResNet50 achieved a mean AUC of 92% (NR), ResNet152 achieved a mean AUC of 93% (NR), and VGGNet16 achieved a mean AUC of 91% (NR). ResNet152, ResNet50, VGGNet16, DenseNet121, InceptionV3, and MobileNetV2 yielded overall accuracies of 77.2%, 74.6%, 71.8%, 67.2%, 63.1%, and 54.8% (NR), respectively.

a Primary metric values are presented as percentages.

b NR: not reported (absolute value) in the original publication.

c CNN: convolutional neural network.

d mAP: mean average precision.

e ViT: vision transformer.

f PU: pressure ulcer.

g CCM: cluster-based classification model.

h BCM: binary classification model.

i PI: pressure injury.

j AUC: area under the curve.

### Synthesis of Results

In total, 40% (6/15) of the studies applied multimodal integration, whereas 60% (9/15) applied unimodal imaging. This review involved 26 models, including AlexNet, VGGNet16, ResNet18, DenseNet121, SE-Swin Transformer, Cascade R-CNN, vision transformer, ConvNeXtV2, EfficientNetV2, Meta Former, TinyViT, CCM, binary classification model, ResNext+wFPN, SE-Inception, Mask-R-CNN, SE-ResNext101, Faster R-CNN, ResNet50, ResNet152, DenseNet201, EfficientNet-B4, YOLOv5, Inception-ResNet-v2, InceptionV3, and MobilNetV2. In total, 46.7% (7/15) of the studies [17,21,23-25,27,28] applied a single model for training, comparing the accuracy of intelligent PI recognition achieved through model application against that of subjective assessments conducted by health care professionals to evaluate model training effectiveness. A total of 40% (6/15) of the studies [15,16,18,19,26,29] applied 2 to 6 models for training, comparing the training outcomes of different models to determine the optimal model capable of achieving the highest accuracy in intelligent PI recognition. In total, 13.3% (2/15) of the studies [20,22] applied multiple models in combination to assess their accuracy in intelligent PI identification. Among all models mentioned, VGGNet16 was applied most frequently, featuring in 20% (3/15) of the studies [15,26,29]. DenseNet121 was applied in 13.3% (2/15) of the studies [15,29], ResNet50 was applied in 13.3% (2/15) of the studies [26,29], and ResNet152 was applied in 13.3% (2/15) of the studies [26,29]. The remaining models were each applied once. CNNs included algorithms such as AlexNet, VGG, ResNet, DenseNet, and EfficientNet. AlexNet, proposed by Krizhevsky et al [30], used a substantial number of parameters, resulting in extended training times and significant computational demands. VGG [31] used small convolution kernels and greater depth but had a large number of parameters and high computational cost. ResNet [32] introduced residual connections, allowing the network to learn identity mappings. DenseNet introduced dense connections, where each layer connects directly to all preceding layers [33]. EfficientNet [34] involved the coordinated scaling of the network’s depth, width, and resolution rather than scaling a single dimension.

### Intelligent PI Identification Training Methods Based on Deep Learning

#### Establishment of a PI Image Database

All studies included in this review that trained intelligent PI identification models first established an image dataset. The number of images in the datasets varied considerably across the included studies, ranging from 395 to 7677. The methods of image acquisition differed. One study obtained them from the Pressure Injury Image Dataset via a public platform [29]. In the study by Swerdlow et al [23], the images were acquired through a specialized company: the PI wound data repository was provided by eKare, which offers professional wound imaging and analysis services. Most studies established a PI image collection by reviewing images of inpatients with PIs and compiling those that met specified criteria.

#### Image Processing

For data augmentation, original PI images underwent flipping, rotation, and cropping to augment the number of baseline samples. For preprocessing, both baseline samples and augmented data underwent image normalization to reduce computational load. Processed images were proportionally divided into training, validation, and test sets.

#### Developing Intelligent PI Identification Models

The final step for the literature included in this review was used to inform model training configuration and human-machine comparisons. Transfer learning was conducted using 26 training models based on the CNN framework. An intelligent PI staging recognition model was developed through deep learning.

### Effectiveness of Intelligent PI Detection Based on Deep Learning

Among the studies included in this review, the accuracy rates for intelligent PI staging identification varied across different models, fluctuating between 54.8% and 93.7%. We present performance figures for illustrative purposes only and do not compare the models’ accuracy. One study indicated that the DenseNet121 model achieved an identification accuracy of 93.7% (not reported [NR]) [15], whereas another study reported an accuracy rate of 67.2% (NR) for the same model [29]. VGGNet16 demonstrated relatively stable accuracy across 20% (3/15) of the studies [15,26,29], achieving values of 83.7%, 82.4%, and 71.8% (NR), respectively. ResNet152 recorded accuracy rates of 84.1% and 77.4% (NR) in 13.3% (2/15) of the studies [26,29]. ResNet50 achieved accuracy rates of 82.9% and 74.6% (NR) in 13.3% (2/15) of the studies [26,29]. The classification performance of AlexNet, VGGNet16, ResNet18, and DenseNet121 yielded overall accuracies of 87.7%, 82.4%, 92.4%, and 93.7% (NR), respectively, with corresponding *F*_1_-scores of 94.7%, 88.2%, 98.2%, and 97.0% (NR) [15]. The SE-Swin Transformer model proposed in the study by Huang et al [16] achieved an accuracy of 87.1% (NR), surpassing other feature extraction models by at least 0.5 percentage points, as well as a mean absolute precision of 80.9% (NR). The results of the study by Cho and Yoo [17] indicated that the PUC-ViT model achieved an accuracy rate of 95.6% (NR) and *F*_1_-score of 95.4% (NR). Among the 4 models applied in the study by Brehmer et al [18]—ConvNeXtV2, EfficientNetV2, MetaFormer, and TinyViT—the transformer-based TinyViT model demonstrated the most effective performance in the binary classification of pressure ulcers, achieving an *F*_1_-score of 93.2% (NR). Among the 2 models applied in the study by Kimura et al [19], the CCM achieved an accuracy of 81.4% (NR), a precision of 81.6% (NR), and an *F*_1_-score of 80.8% (NR). The binary classification model attained an accuracy of 87.1% (NR), a precision of 84.1% (NR), and an *F*_1_-score of 85% (NR). The study by Wang et al [20] increased the accuracy rate for stage 3 PI diagnosis from 60.3% to 76.2% (NR) using ResNext+wFPN. The *F*_1_-scores for stage 1 to 4 PI diagnosis were 81.5%, 80.8%, 81.6%, and 81.1% (NR), respectively. In the study by Gui et al [21], the SE-Inception model achieved an accuracy of 93% (NR) and an area under the curve of 94% (NR). In the study by Zalluhoğlu et al [22], a semiduplex CNN achieved an accuracy of 93% (NR) and *F*_1_-score of 93% (NR). In the study by Swerdlow et al [23], the Mask-R-CNN model achieved an overall classification accuracy of 92.6% (NR) and a segmentation accuracy of 93.0% (NR). For stages 1 to 4, the model obtained *F*_1_-scores of 84.2%, 94.7%, 90.7%, and 94.4% (NR) and Dice coefficients of 92%, 85%, 93%, and 91% (NR), respectively. In the study by Kim et al [24], the model achieved top-1 accuracy scores of 79.3% and 71.7% (NR) and *F*_1_-scores of 79.1% and 71.5% (NR) on the internal and external test sets, respectively. The study by Fergus et al [25] used a confidence score threshold of 75% (NR), yielding an average precision of 67.9% and *F*_1_-score of 67.8% (NR). In the study by Seo et al [26], for EfficientNet-B4, the macro–*F*_1_-score was calculated to be 89.4% (NR), and the average performance of 2 experienced nurses was reported as 87.8% (NR). EfficientNet-B4 achieved an accuracy of 91.4% (NR). In the study by Aldughayfiq et al [27], the YOLOv5 model achieved an overall mean average precision 50 of 76.9% (NR) on the validation set, enabling it to accurately detect and classify PIs with a high degree of confidence. In the study by Liu et al [28], the Inception-ResNet-v2 model achieved an accuracy of 98.5% and an *F*_1_-score of 94.1% (NR). In the classification task of necrotic tissue, the model attained an accuracy of 97% and an *F*_1_-score of 93.5% (NR). Finally, in the study by Ay et al [29], ResNet152, ResNet50, VGGNet16, DenseNet121, InceptionV3, and MobileNetV2 achieved overall accuracies of 77.2%, 74.6%, 71.8%, 67.2%, 63.1%, and 54.8% (NR), respectively, with corresponding mean area under the curve values of 93%, 92%, 91%, 91%, 85%, and 85% (NR), respectively.

### Mapping the Translational Landscape: From Retrospective Development to Clinical Validation

**Figure 2.**
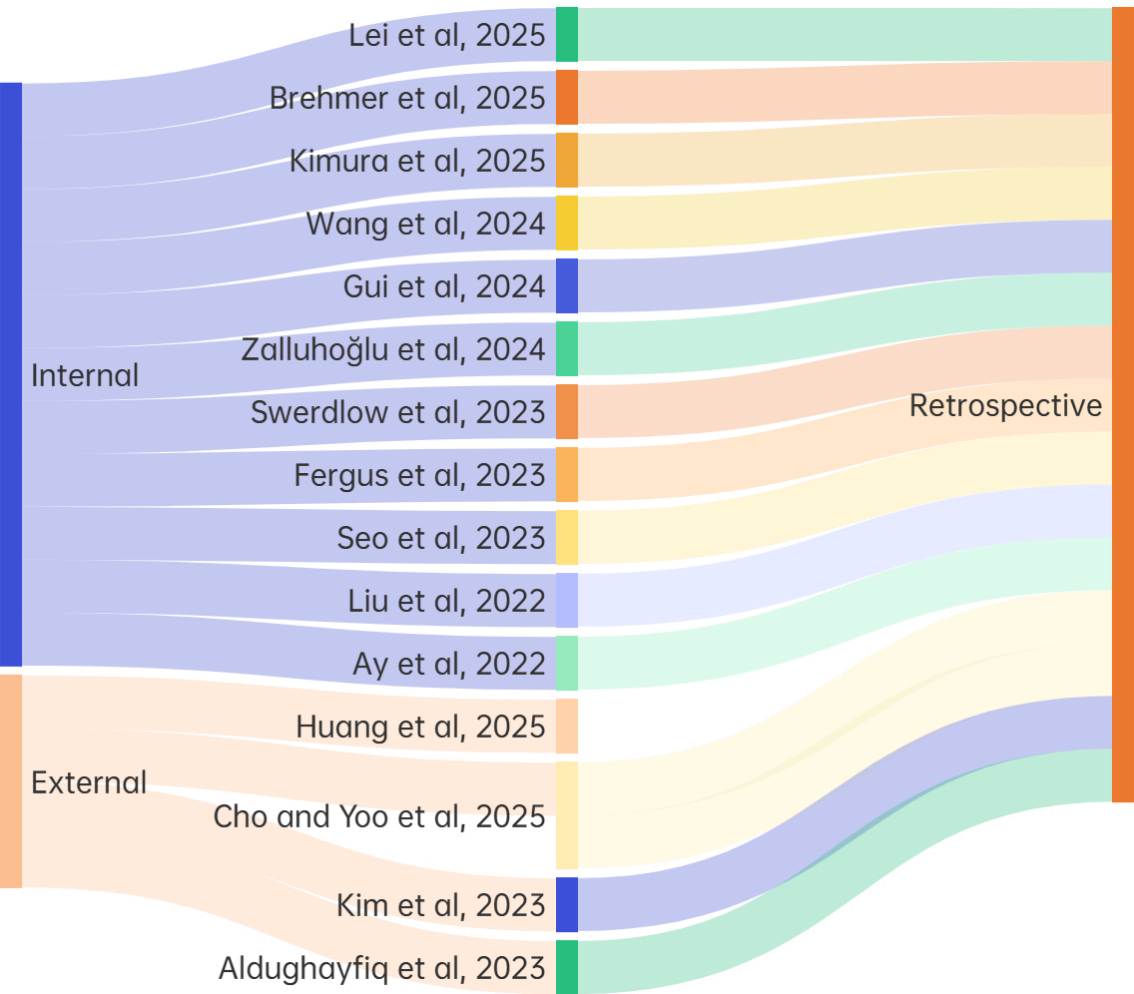
Validation Sankey diagram [15-29].

The validation Sankey diagram (Figure 2 [15-29]) delineated a critical attrition curve, revealing that while all 15 studies had retrospective designs, the flow of evidence was sharply constricted at the validation checkpoint, with only 26.7% (4/15) incorporating external test sets. For the purpose of this review, “external validation” was broadly defined to include any testing on data not used during model training, “multicenter” was defined as testing on data from a physically different institution from those of the training data, and “public dataset” was defined as testing on a publicly available benchmark dataset distinct from the authors’ private training data.

**Figure 3.**
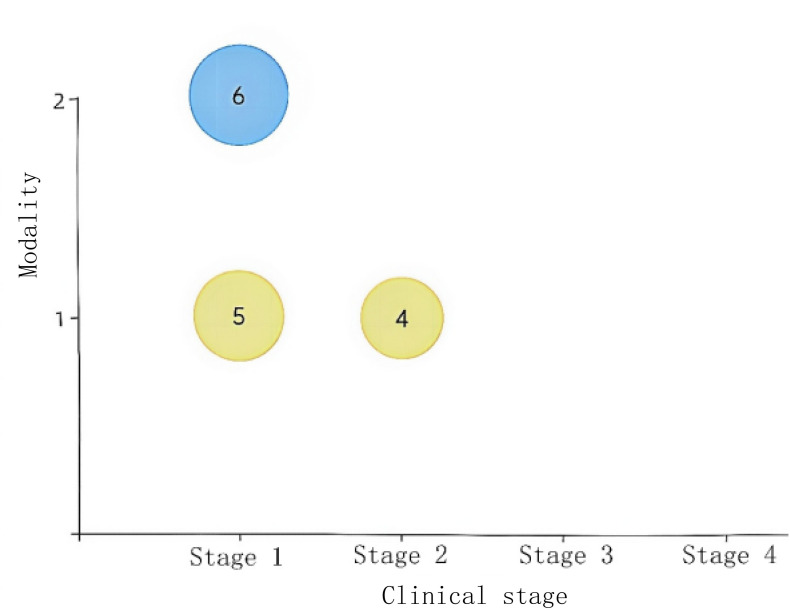
Grouped bubble chart of data modality vs clinical stage illustrating the distribution of the included studies (N=15). The x-axis represents the clinical stage of algorithm deployment: stage 1 corresponds to algorithm development, stage 2 corresponds to technical validation, stage 3 corresponds to workflow integration, and stage 4 corresponds to clinical trial. The y-axis categorizes studies by data modality: unimodal (1) vs multimodal (2). The bubble size is proportional to the number of studies, whereas bubble color denotes the modality type (blue for multimodal; yellow for unimodal).

This bottleneck was further elucidated by the grouped bubble chart (Figure 3), which mapped the intersection of modality and clinical stage. While most of the research (11/15, 73.3%) remained concentrated at stage 1 (development), a notable finding was that 54.5% (6/11) of the studies in stage 1 were multimodal. This suggested that while the field was dominated by retrospective single-modality development, multimodal integration appeared to be a distinguishing characteristic of the studies that will progress toward clinical validation.

## Discussion

### Summary of Evidence

This scoping review mapped the contemporary landscape of deep learning in PI identification, revealing a field characterized by a profound modality chasm between computational sophistication and clinical utility. While the evolution of unimodal imaging exhibits diminishing returns, with marginal performance gains despite increasing model complexity, the integration of multimodal data remains nascent and fragmented.

#### Accuracy of Intelligent PI Identification Models: Generally High, With Variations Observed

On the basis of the results of the studies included in this review, the DenseNet121 intelligent model achieved an identification accuracy of 93.7%. However, in another study, an accuracy of 67.2% was reported for the same model. The former was a unimodal study, whereas the latter was a multimodal one. Although the former demonstrated high accuracy in internal validation, it was inherently limited by the physical constraints of visible light and lacked the physiological context necessary to distinguish ischemic tissue or assess the condition of subcutaneous tissue. The cross-study comparisons of accuracy are not valid for selecting a single best model. Owing to the lack of standardized benchmark tests, it is not possible to compare accuracy rates across different datasets, class definitions, and levels of data annotation accuracy. One must also consider multiple factors. First, there was a difference in dataset size. The study in which high accuracy was achieved used a larger dataset comprising 7677 images, whereas the study with lower accuracy used 1091 images. This disparity prevented the model from adequately learning features. In a study by Wang et al [35], an intelligent burn depth recognition model was established using 5637 complete burn wound images to achieve satisfactory recognition results. Therefore, future research can increase the size of the dataset to enhance the sample balance and obtain more precise outcomes. Second, the class definitions differed significantly. Studies that achieved high accuracy used stratified sampling to maintain category proportions, whereas those with low accuracy used random splitting, which resulted in inconsistent distributions between the test and training sets. For instance, the training set may predominantly feature mild injuries, whereas the test set may contain mostly severe ones [36]. Consequently, future researchers should clearly delineate the image datasets to ensure a balanced category distribution in the test set. Third, regarding data annotation accuracy, studies with better performance tend to employ larger and more specialized annotation teams—in the most rigorous datasets, including five professionals—while the studies with poorer performance more often rely on smaller teams, such as two professionals. This may lead to inconsistent annotations for visually ambiguous cases. This underscores the necessity for at least 3 professionals to perform normative annotation in future studies, thereby reducing ambiguity [37]. The research methodology of the primary studies included in this scoping review and process were meticulously executed, with a well-structured experimental design [29]. Although DenseNet121 exhibited lower recognition accuracy, ResNet50 demonstrated superior performance, suggesting that the experimental design may be more suited to training ResNet50. It is recommended that future research designs focus on the design and training of a single model. Clinical AI systems should not be judged on technical performance alone but also on clinically meaningful utility [38]. Images cannot quantify blood flow. For instance, distinguishing a stage 1 PI (nonblanchable erythema due to occlusion) from healthy, well-perfused skin is notoriously difficult through color alone. Thermal imaging is required to visualize temperature gradients and perfusion changes. The viability of granulation tissue depends on oxygen saturation. Hyperspectral or spatial frequency-domain imaging is necessary to measure hemoglobin oxygenation and water content. The path to clinical-grade reliability lies in fusing these heterogeneous data sources. Future PI research should focus on tangible clinical benefits. A notable finding of this review is that, despite the availability of more advanced architectures, VGGNet16 remained widely adopted. This suggests that researchers tend to favor established baseline models over architectural innovation. Although VGGNet16 provides a stable benchmark, its inherent limitations—particularly its large parameter size and lack of multi-scale feature integration capabilities—likely constrain the potential of multimodal systems [39].

#### Application of Intelligent PI Identification Models Demonstrates Substantial Clinical Value

Of the 15 included studies, 2 (13.3%) compared the models with human professionals [18,26]. Regarding the task of staging PIs, only one of these studies indicated that deep learning models achieved a lower identification accuracy than nurses with 19 years of experience [26]. On the basis of the evidence, valid benchmarking should only occur under the following specific circumstances: stratified by experience level (eg, novice nurses, general ward nurses, and certified wound care specialists), simulated time constraints, rigorous blinding, and input parity. In both accuracy and speed, the other models (ResNet152, VGGNet16, and ResNet50和DenseNet201) outperformed nurses with varying years of experience. For less experienced health care personnel, accurately staging PIs remains challenging, potentially influenced by inadequate training, information gaps, observer bias, and the diverse appearance of wounds [40]. The skin tone, age, and health status of the patient may also interfere with visual assessment [41]. Intelligent recognition models can rapidly learn from medical data, accumulating within a short time frame the expertise that a wound specialist might gain over months or even years. Moreover, computers process data using mathematical algorithms that make probabilistic judgments based on objective rules, ultimately delivering results. This approach solves issues such as the inability to quantify PI staging and the lack of objectivity in diagnosis [29]. In addition, wound healing monitoring can be enhanced through a thermal imaging–based smartphone app, which provides a related example of technology-enabled wound assessment and may help position PI AI within a wider digital wound care landscape [42].

### The Imperative for Multimodal Integration: Beyond Visual Confirmation

The notable scarcity of true multimodal studies identified in this review underscores the next frontier for research. Our findings suggest that meaningful transition requires the integration of heterogeneous data sources. Currently, existing multimodal attempts are limited to incorporating thermal or basic clinical data. However, to bridge the translational gap, future systems must evolve from superficial sensor fusion to deep multimodal integration. This requires synergizing imaging data with longitudinal electronic health record, nutritional biomarker, and mobility data to capture a holistic view of patient health [43].

### Limitations

A limitation of this scoping review is the absence of a formal methodological quality appraisal of the included studies. According to standard scoping review methodology, the focus was on mapping the landscape of available evidence rather than assessing the risk of bias or the certainty of evidence.

### Conclusions

The current landscape of deep learning in PI identification is heavily skewed toward unimodal imaging, creating a modality gap that hinders clinical translation. While unimodal models demonstrate high accuracy in controlled settings, they lack the physiological context required for robust wound assessment. Future research must prioritize bridging the gap from unimodal imaging to true multimodal integration, shifting the focus from algorithmic architecture to the fusion of heterogeneous data sources to achieve clinical-grade reliability.

## Supplementary material

10.2196/91048 Multimedia Appendix 1 Eligibility criteria.

10.2196/91048 Multimedia Appendix 2 Search strategy.

10.2196/91048 Checklist 1 PRISMA-ScR checklist.

## Abbreviations

CNN: convolutional neural network
NR: not reported
PI: pressure injury
PRISMA-ScR: Preferred Reporting Items for Systematic Reviews and Meta-Analyses extension for Scoping Reviews
