# Supplementary material for: Unimodal and Multimodal Deep Learning for Pressure Injury Identification: Scoping Review
Source: JMIR Med Inform. 2026 Aug 10;14:e91048. doi: 10.2196/91048 (PMC13456529; doi:10.2196/91048)
Supplement: Supplementary file 1 [file medinform-v14-e91048-s001.docx]

**Appendix 1. Eligibility criteria.**

|  | **Inclusion** | | **Exclusion** |
| --- | --- | --- | --- |
| **Population** | Patients with pressure injuries (PIs). Covering all age groups and clinical settings (ICU, long-term care, home care). | | Literature focusing primarily on non-pressure-related injuries (such as burns, acute trauma and skin cancer). |
| **Concept** | Intelligent Recognition: Using Deep Learning (DL), such as:  Detection  Classification/Grading  Segmentation  Unimodal studies  Multi-modal: Must integrate two or more data modalities, such as:  Visual (RGB) + Depth  Visual (RGB) + Thermal  Visual + Clinical text/Electronic Health Records (EHR)  Visual + Audio/Sensor data. | | Studies that use AI solely for prognostic predictions (such as predicting healing times) without any identification or diagnostic components. |
|  | |  |  |
| **Context** | Clinical settings (hospitals, clinics, long-term care facilities).  Telemedicine/home care settings | | Pure theoretical research on algorithms not involving clinical applications. |
| **Type of source** | Original research articles, theses/dissertations, and conference papers, published up to April 2026.  English language studies will be included. | | Reviews, case reports, preprints, study protocols, conference abstracts, posters, editorials, and commentaries. |
