# Supplementary material for: Unimodal and Multimodal Deep Learning for Pressure Injury Identification: Scoping Review
Source: JMIR Med Inform. 2026 Aug 10;14:e91048. doi: 10.2196/91048 (PMC13456529; doi:10.2196/91048)
Supplement: Supplementary file 2 [file medinform-v14-e91048-s002.docx]

**Appendix 2 Search Strategy**

PubMed

| Search | Query |
| --- | --- |
| #1 | "Pressure Ulcer"[MeSH Terms] |
| #2 | (((("Wounds and Injuries"[Title/Abstract]) OR ("Pressure Injur*"[Title/Abstract])) OR ("Wound*"[Title/Abstract])) OR ("Pressure Sore*"[Title/Abstract])) OR ("Ulcer*"[Title/Abstract]) |
| #3 | #1 OR #2 |
| #4 | (("Deep Learning"[MeSH Terms]) OR ("Machine Learning"[MeSH Terms])) OR ("Neural Networks, Computer"[MeSH Terms]) |
| #5 | ((((((((((("Convolutional Neural Networks"[Title/Abstract]) OR ("Multimodal Imaging"[Title/Abstract])) OR ("Multi-modal"[Title/Abstract])) OR ("Fusion"[Title/Abstract])) OR ( "Machine Learning"[Title/Abstract])) OR ("AI"[Title/Abstract])) OR ( "Neural Network*"[Title/Abstract])) OR ("deep learn*"[Title/Abstract])) OR ("convolutional neural network*"[Title/Abstract])) OR ("cnn"[Title/Abstract])) OR ("artificial intelligence"[Title/Abstract])) OR ("machine learn*"[Title/Abstract]) |
| #6 | #4 OR #5 |
| #7 | #3 AND #6 |
| #8 | "Diagnostic Techniques and Procedures"[Mesh Terms] |
| #9 | (((("Classification"[Title/Abstract]) OR ("Detection"[Title/Abstract])) OR ("Diagnosis"[Title/Abstract])) OR ("Identification"[Title/Abstract]) OR ("Staging"[Title/Abstract]) |
| #10 | #8 OR #9 |
| #11 | #7 AND #10 Filter: English |

Cochrane Library

| Search | Query |
| --- | --- |
| #1 | ("Wounds and Injuries"):ti,ab,kw OR ("Pressure Injur*"):ti,ab,kw OR ("Wound*"):ti,ab,kw OR ("Pressure Sore*"):ti,ab,kw OR ("Ulcer*" OR "Skin Ulcer"):ti,ab,kw |
| #2 | MeSH descriptor: ["Pressure Ulcer"] explode all trees |
| #3 | #1 OR #2 |
| #4 | ("Convolutional Neural Networks" OR "Multimodal Imaging" OR "Multi-modal" OR "Fusion" OR "convolutional neural network*" OR "Neural Network*"):ti,ab,kw OR ("Machine Learning" OR "deep learn*" OR"machine learn*" OR "deep Learning"):ti,ab,kw OR ("artificial intelligence" OR "AI"):ti,ab,kw OR ("cnn") :ti,ab,kw OR ("Fusion"):ti,ab,kw |
| #5 | MeSH descriptor: ["Deep Learning"] explode all trees |
| #6 | MeSH descriptor: ["Machine Learning"] explode all trees |
| #7 | MeSH descriptor: ["Neural Networks, Computer"] explode all trees |
| #8 | #4 OR #5 OR #6 OR #7 |
| #9 | MeSH descriptor: ["Diagnostic Techniques and Procedures"] explode all trees |
| #10 | ("Classification"):ti,ab,kw OR ("Detection"):ti,ab,kw OR ("Diagnosis"):ti,ab,kw OR ("Identification"):ti,ab,kw OR ("Staging"):ti,ab,kw |
| #11 | #9 OR #10 |
| #12 | #3 AND #8 AND #11 Filter: English |

Web of Science

| Search | Query |
| --- | --- |
| #1 | ((((TS=("Pressure Ulcer")) OR TS=("Wounds and Injuries")) OR TS=("Pressure Injur*")) OR TS=("Pressure Sore*")) OR TS=("Ulcer*") |
| #2 | ((((((((((((((TS=("Deep Learning")) OR TS=("Machine Learning")) OR TS=("Neural Networks, Computer")) OR TS=("Convolutional Neural Networks")) OR TS=("Multimodal Imaging")) OR TS=("Multi-modal")) OR TS=("Fusion")) OR TS=("Machine Learning")) OR TS=("AI")) OR TS=("Neural Network*")) OR TS=("deep learn*")) OR TS=("convolutional neural network*")) OR TS=("cnn")) OR TS=("artificial intelligence")) OR TS=("machine learn*") |
| #3 | (((((TS=("Diagnostic Techniques and Procedures")) OR TS=("Classification")) OR TS=("Detection")) OR TS=("Diagnosis")) OR TS=("Identification")) OR TS=("Staging") |
| #4 | #1 AND #2 AND #3 Filter: English |

IEEE Xplore

| Search | Query |
| --- | --- |
| #1 | (((("Full Text & Metadata"=("Pressure Ulcer")) OR "Full Text & Metadata"=("Wounds and Injuries")) OR "Full Text & Metadata"=("Pressure Injur*")) OR "Full Text & Metadata"=("Pressure Sore*")) OR "Full Text & Metadata"=("Ulcer*") |
| #2 | (((((((((((((("Full Text & Metadata"=("Deep Learning")) OR "Full Text & Metadata"=("Machine Learning")) OR "Full Text & Metadata"=("Neural Networks, Computer")) OR "Full Text & Metadata"=("Convolutional Neural Networks")) OR "Full Text & Metadata"=("Multimodal Imaging")) OR "Full Text & Metadata"=("Multi-modal")) OR "Full Text & Metadata"=("Fusion")) OR "Full Text & Metadata"=("Machine Learning")) OR "Full Text & Metadata"=("AI")) OR "Full Text & Metadata"=("Neural Network*")) OR "Full Text & Metadata"=("deep learn*")) OR "Full Text & Metadata"=("convolutional neural network*")) OR "Full Text & Metadata"=("cnn")) OR "Full Text & Metadata"=("artificial intelligence")) OR "Full Text & Metadata"=("machine learn*") |
| #3 | ((((("Full Text & Metadata"=("Diagnostic Techniques and Procedures")) OR "Full Text & Metadata"=("Classification")) OR "Full Text & Metadata"=("Detection")) OR "Full Text & Metadata"=("Diagnosis")) OR "Full Text & Metadata"=("Identification")) OR "Full Text & Metadata"=("Staging") |
| #4 | #1 AND #2 AND #3 Filter: English |
